# On the Pathology of the Blood

**Published:** 1831

**Authors:** 


### On the Pathology of the Blood. By *M. Andral.*

[Pathological Anatomy, Vol. I.]

The important subject at the head of this article occupies fifty pages of the first volume of M. Andral's work, as translated by Drs. Townsend and West—and is well deserving, we think, of a separate article in this Journal. We have always been of opinion that, whatever be the *origin* of a vitiated condition of the blood and other fluids of the body, this morbid state plays a very important part in the subsequent enactment of disorder in the constitution. The investigation is, therefore, extremely interesting, and of a highly practical nature. Even when the doctrine of solidism was almost exclusive in France, the immortal Bichat observed that, although the humoral pathology had, no doubt, been carried too far in former times, yet there was no doubt that it was founded in truth—and that, "in a great many cases, we must allow that *all* should be referred to morbid humours."* Bichat did not follow up this idea in any of his works, nor did any of his contemporaries act on the suggestion. The extravagances of the humoral theorists, and the dangerous practice to which such extravagances led, put rational practitioners on their guard against the humoral pathology—and this doctrine appeared to be consigned to oblivion. But it was easy to foresee, that exclusive solidism would be as inadequate to explain the phenomena of disease as exclusive humoralism; and, therefore, M. Andral acted a judicious and useful part in collecting facts, and drawing up a kind of inventory of all we are in possession of that bears on the subject under consideration. Let us, says he, endeavour to determine accurately where we are, in order that we may know where we are going—to what conclusion we are proceeding—and how we are to arrive at it.

The alterations of the fluids should be studied—*first,* in the blood—*secondly,* in the different humours concurring to form the blood, or emanating from that fluid. The qualities of the chyle and lymph must have a direct influence on the blood—and if we find the secretions modified in quality or quantity, we may often infer modifications in the state of the blood whence they are derived.

"The blood, while circulating, seems to be under the influence of two forces. The one imparts to the mass an intestine motion, by virtue of which each of its globules moves on by itself, surrounded by an envelope of co-

---

* Introduction to his "Anatomie Générale."

louring matter, and keeping at a certain distance from the rest. This double action of attraction and repulsion ceases to take place the moment the blood leaves its vessels. The other force, directly opposite to the preceding, tends to bring the blood to a state of repose: it is exerted in the organic parenchymas at the point of contact of the solids and the blood. The blood, when examined with a microscope in these parenchymas, has been compared to a kind of whirlpool, from which particles were incessantly detached to be lost in the solid substance, whilst others were quitting this latter and returning into the vortex. If, then, there is a great difference between the blood contained in the large vessels and the different solids, that is by no means the case in the capillaries; in these, the blood and the tissues to which it is distributed tend to be confounded together. At their point of contact, the blood assumes the nature of the solid, and becomes organized; so that its vitality is no longer dubious. But it is not there only that we can discover a power of organization in the blood; we find it active and prolific wherever the fibrine coagulates, whether in the vessels or out of them. I have already shewn how, under such circumstances, vessels make their appearance in the fibrine, how a circulation becomes established, secretions take place, and tissues are developed in it. If we analyze the blood and the solids, we discover the same proximate principles in both. If we examine their physical structure, we find it identical, both consisting of globules mixed with an amorphous substance. Bordeu acknowledged this identity of composition when he said that blood is fluid flesh, '*Le sang est de la chair coulante.*'

Thus, then, in the three-fold respect of the vital phenomena, intimate structure, and chemical composition, no line of demarcation can be drawn with strictness and precision between the blood and the solids. Physiologically speaking, it is impossible to conceive that one of these two parts of the same whole could be modified without the other being so likewise. On the one hand, inasmuch as the blood nourishes the solids, and as without its presence they cannot support life, the state of the solids cannot but be influenced by the state of the blood. The chemist might as well say that the nature of a body does not depend on the nature of the elements that compose it. On the other hand, the solids, considered with respect to their relations to the blood, form but two classes: the one contributing to *make* the blood, such as those concerned in the actions of absorption, digestion, arterial circulation, and respiration; the other contributing to *unmake* it, those, namely, concerned in the actions of venous circulation, secretion, and nutrition. No one solid, therefore, can undergo the slightest modification, without producing some derangement in the nature or quantity of the materials destined to form the blood, or to be separated from it. Physiology, then, leads us to the conclusion that every alteration of the solids must be succeeded by an alteration of the blood, just as every modification of the blood must be succeeded by a modification of the solids. Viewed in this light, there is no longer any meaning in the disputes between the solidists and the humorists; the system appears to constitute but one great whole, indivisible in the state of health as well as in that of disease; the division of the parts of the body into solids and fluids seems to be a distinction of small importance, and one that is not always just, since it ceases to exist in the intimate structure of the organs, in which all the grand vital phenomena take place, and in which, also, occur all the changes that constitute the morbid state." 643.

The intimate and necessary dependence, then, of the fluids on the solids, and the solids on the fluids, being acknowledged, how are we to proceed? We must carefully observe the facts of changes in the blood and its products, and note the phenomena, drawing, when we can, such conclusions as seem warranted by the data. Chemistry informs us that the blood is composed of fibrine, albumen, a peculiar colouring matter, free soda, oxide of iron and lime, with a number of different salts, as lactate of soda, muriate of soda and potash, phosphate of soda, &c. &c. &c. besides some other peculiar matters of recent discovery, as carbonic acid, oily matter, urea, &c. Thus the elements of most of the organs of the body, and of some of the secreted fluids, are found in the blood itself. Observation has long made us familiar with the separation of the blood, when drawn from vein or artery, into two parts—the *solid*, principally composed of fibrine and colouring matter—the *fluid*, consisting chiefly of water and albumen. The author then goes on to study the pathological conditions of the blood in respect to each of the elements abovementioned.

"The fibrine may be altered either in quantity or in quality. In the first place, there are cases in which this principle is more abundant than usual, or at least in greater proportion relatively to the water and albumen. In such cases, the blood, when drawn from a vein, forms in the vessel that receives it a clot with little or no serum. These are however to be divided into two classes. In the first class of cases, the fibrine constituting the clot, still contains a pretty large quantity of serum, which may be separated from it by pressure; in these the coagulum has but little density. In the second, on the contrary, the clot is very dense, and a little fluid albumen can with difficulty be squeezed out of it. In the first class the relative increase of quantity of the fibrine is only apparent; in the second, it is real. We must take care not to confound them, as they belong to different states of the system. The very fibrinous blood is commonly called *rich blood:* it may either depend simply on a vigorous constitution, or on certain morbid states.

In place of being superabundant in the blood, the fibrine may, on the contrary, be quite the reverse. There are, in fact, some persons whose blood, when taken from a vein, presents but a very small coagulum in proportion to the large quantity of serum in which it appears. But here, likewise, we must make a distinction. The diminution of quantity of the fibrine may be only apparent; as happens when its particles, being very strongly condensed, are much closer to each other than in their natural condition; the clot is then very small, and remarkably firm; this occurs very often, for instance, in patients suffering under acute rheumatism. In other cases, the clot is not only very small, but also very soft; and there is then really a deficiency of fibrine. This may be observed in many cases of chronic disease, or in persons with a slender muscular system, and habitually pale skin." 646.

Thus we see that the proportions of fibrine in the blood vary considerably, and we have reason to know that the constituent principles of the fibrine itself also vary, and in ways which we can hardly expect to ascertain by chemical analysis, though we may well believe that such variations will operate on the functions of the living machine.

"The force that tends to keep the globules of fibrine at a certain distance from each other during life, may be so modified as that they shall have a tendency to run together, as they naturally do after death; and hence may result the spontaneous coagulation of the blood in its vessels during life. There have been too many cases of this nature observed, for us of the present day to attempt to deny the possibility of its occurrence. Sometimes it takes place without any known cause; and sometimes it appears to accompany a state of irritation in the parietes of the containing vessel. When the blood once becomes solid, it displays indubitable symptoms of vitality; vessels are produced, and secretions formed in it; and different alterations of nutrition, resembling those observed in the tissues, may also occur. If we examine whence this coagulated blood derives its vitality, we find that it cannot partake of the common life of the rest of the body, since it very often merely touches the surrounding tissues, without being in any manner continuous with them. We must therefore admit that these polypiform concretions, or *polypi*, as they are called, may possess a proper vitality, by means of organs they have created themselves." 648.

In other cases, on the contrary, there is a diminution of force in the cohesion of the fibrinous particles—hence a less tendency of the blood to coagulate—or a total absence of coagulum—or when there is a coagulum, it is soft, and void of resistance. In some cases there is no appearance of even the fibrinous particles, which are completely mixed with the serum, producing a fluid mass, of a reddish or black colour. These different appearances produced in venesection, may also be seen in the dead body. The white layer of coagulum, known by the name of inflammatory buff, is well understood.

"The buffy coat is formed of pure fibrine, with which is mixed a certain quantity of serum, which, according to the researches of Dauler and of Gendrin, contains much more albumen than the serum of the rest of the blood. The greatest analogy exists, both in respect of appearance and of chemical composition, between the buffy coat of the blood and the substance that constitutes the false membranes of the serous cavities." 650.

The experiments of Dr. Traill, confirmed by M. Gendrin, prove that in inflammation, the serum of the blood contains nearly twice as much albumen as in the healthy state. This increase of the albumen may be detected simply by the touch—the serum being then remarkably viscid. In other cases, on the contrary, the very small coagulum obtained by heating the serum (the greater part of which evaporates) proves how much the quantity of the albumen is diminished.

" It is worth while remarking, in many of these cases, how very just are the common expressions of *impoverished blood, watery blood, blood turned into water, &c.*

In some persons, the blood drawn from a vein is remarkable for the intensity of its colour; in others, on the contrary, it is exceedingly pale, the coagulum is of a rose-coloured white, and the serum resembles water; in this case the colouring matter of the blood is either diminished in quantity, or altered in its nature. This in general co-exists with an increase of the serous part of the blood. The human being, in such cases, appears in this respect to descend in the zoological scale, and his blood tends to become analogous to the naturally colourless blood of certain animals. The same causes that we have already seen producing anæmia, are generally those also which tend to diminish the quantity of the peculiar animal matter to which the blood owes its colour." 652.

In another part of the work, M. Andral has mentioned cases where there were discovered in the blood not only different elements of the secreted fluids, but also sundry morbid productions, such as pus, encephaloid matter, entozoa, and calculous concretions. In whatever way these have originated, there can be no doubt that they change the constituency of the circulating mass—and vitiate the living current.

" Now, who could assert that in such cases the blood is not deeply altered in its nature. Besides, most commonly there are at the same time in the texture of many of the solids, morbid secretions, purulent or otherwise, formed of a matter that has the greatest analogy to that found in the vessels. Amongst the cases of this description I have had an opportunity of observing, I shall adduce the following. In a woman who died at *La Charité* with all the symptoms of a chronic affection of the lungs and of the digestive passages, I found in front of the vertebral column an enormous tumour composed of an agglomeration of lymphatic ganglions, which, instead of their natural tissue, presented merely an inorganic pap, of a greyish or reddish colour. A similar substance appeared in the liver, in the form of roundish isolated masses; it was also found in the spleen, in the cells of which it appeared to be deposited in place of the blood they usually contain; lastly, in several points of the lungs, the lobules were infiltrated with the same substance. But that was not all; for in both lungs, a great number of the branches of the pulmonary artery contained, instead of blood, a curdy matter of a reddish grey colour, resembling in appearance the morbid matter found in the mesenteric ganglions, liver, spleen, and lungs. The right cavity of the heart, the pulmonary artery, and its first divisions, contained a blood of little colour, and poor consistence. In another woman, who had a broken down cancer of the uterus, all the veins of that organ, and the trunk of the vena cava up to its passage under the liver, were full of a semifluid sanious matter of a greyish or reddish white. In a man far from being advanced in life, who had in a great many organs, encephaloid masses in a state of softening, the inferior vena cava, the renal and splenic, and some branches of the superior hepatic vein and of the pulmonary vessels, were filled with a kind of detritus of a reddish grey colour, without its adhering to the venous parietes, which, in this case, as well as in the preceding cases, presented no appreciable trace of alteration. Facts similar to those just mentioned have also been observed by others. Thus, Beclard mentions a case in which the heart and principal trunks of the vessels were filled with a solid clot, the interior of which presented numerous collections of encephaloid matter. M. Velpeau found a mass of encephaloid in the midst of a clot of blood contained in the vena cava. He also cites a case of a man that died almost suddenly, after having shewn some symptoms of cerebral congestion, and in whom, upon examination, there was found through the whole extent of the circulatory system, a blood of a pultaceous consistence and blackish red colour, resembling the matter of certain abscesses of the liver.

Bichat, in his *Anatomie Génèrale*, has related a case in which the vena portæ and the hepatic and splenic veins, instead of blood, were filled even to their very last ramifications with a greyish sanies. I have already brought forward cases in which a matter exactly similar to pus was found in the midst of a clot in one of the cavities of the heart, without any purulent collection existing elsewhere in the body." 656.

The alterations of the blood are indicated by other means than simple inspection or chemical analysis. We know that, in general, the blood of one animal may be transfused into the vessels of another, with impunity. When we find that the blood of a diseased animal, when introduced into the circulation of another, proves a real poison, it is impossible not to conclude that the nature of the blood is changed in such diseased state. The following facts are deserving of record.

" M. Gendrin, in his work on fevers, (vol. ii. p. 145,) gives an account of a flayer whom he attended in a putrid fever with an eruption of gangrenous pustules. An ounce of blood drawn from one of the patient's veins was injected into the cellular tissue of the groin of a cat. The consequences to the poor animal were copious vomitings of bile, at first yellow, and then greenish, dyspnœa, a small, frequent, and irregular pulse, a dry and brown tongue, a constantly increasing prostration of strength, and, towards the close of the scene, some slight convulsive motions at intervals. Death ensued in six hours and fifty minutes after the injection. The appearances observed on examining the body are described as follows:—The skin of the groin did not adhere to the subjacent parts; the cellular tissue was soft and almost pulpy, and of an ashy yellow colour; it exhaled a fetid odour, and was dotted with small red spots; the gastro-mucous membrane of the stomach and intestines was in the natural state, that of the respiratory passages was of a reddish brown; the lungs, especially the left, contained black blood, and were full of browish black spots; the blood throughout the whole body was black and fluid; in the left pleura were about two ounces of very serous black blood; the heart was soft and flaccid; there was no appearance of lesion in the brain or spinal marrow; the body speedily began to exhale a fetid odour.

Some blood that proceeded from an epistaxis that occurred in the same patient, was injected into the femoral vein of a dog. The animal exhibited the same series of symptoms as the one in the preceding experiment, which, in like manner, terminated in death.

In another work, (*Histoire des Inflammations*, vol. ii.) M. Gendrin relates some experiments in which he injected into the veins of animals the blood of persons labouring under confluent small-pox. Very severe symptoms, which rapidly proved fatal, ensued; and on opening the bodies, several organs were found in a state of high inflammation.

M. M. Dupuy and Leuret introduced into the cellular tissue and veins of a sound horse, blood that came from horses affected with malignant anthrax, ('charbon') and thus succeeded in producing the disease. It is, then, beyond all doubt, that in this case the blood itself was altered in its nature, since it proved capable of transmitting the affection.

These facts bring to our recollection some others, for an account of which we are indebted to the celebrated Duhamel. He has related a case where an ox, that was over-driven, having been slaughtered at an inn at Pithiviers, the butcher put into his mouth, for a few moments, the knife he had employed for the purpose. The consequence was, that in some hours afterwards his tongue swelled, his breathing became difficult, and then blackish pustules broke out all over his body: at the end of four days he died. The inn-keeper wounded himself with a bone of the same ox in the palm of his hand; his arm mortified, and he died in seven days. Two women having received some drops of the blood of the same animal, the one on her hand, the other on her cheek, these parts were seized with a gangrenous inflammation. Is it not likewise the simple contact of the blood of diseased animals that produces malignant pustule in man?

From these facts we must conclude that, under certain circumstances, the blood may be altered in its intimate nature, so as to acquire noxious properties, which display themselves when it is mixed with the blood of healthy animals." 659.

The alterations in the blood hitherto treated of, may be ascertained by experiment; but there are others which, M. Andral thinks, we might fairly admit from induction. Thus, for instance, if an individual has breathed an atmosphere impregnated with deleterious miasmata, or feeds on unwholesome food, and becomes sick in consequence, physiology would lead us to infer that the blood, in such a case, "has been at least the vehicle of the morbific matter residing in the air or in the food." If bad diet furnish bad chyle, we may reasonably conclude that this chyle will not form good blood.

" But when we inject into the veins of animals different organized substances in a state of putrefaction, the blood, in such a case, is not merely a vehicle to carry to the solids the deleterious substances that inflame them; its unusual appearance leaves no room to doubt its being really altered in its nature; thus, it readily putrefies, it has lost the power of coagulating, the force of aggregation uniting its molecules is singularly diminished, and the most of the tissues become like filtres, through which it oozes on every side. Various animal poisons, such as those of several of the snake tribe, and different mineral poisons, as mercury, for instance, act upon the blood in the same manner." 660.

There are some supposed or real electrical changes in the blood during disease, but their nature is not exactly ascertained. The direct influence of the nerves on the blood must be taken into consideration. In the great vessels, indeed, where the stream is large and rapid, we cannot suppose that much influence can be excited by the nerves dispersed over the internal surface of the vessels; but in the capillaries, where the blood comes into contact with the solids, and is, as it were, confounded with them—where it manifests signs of vitality—and where, in conjunction with the nerves, it gives life to the organs which it traverses—in these, it would be difficult to deny the influence of the nerves on the blood. It is in the capillaries that the great law of mutual dependence, which connects all the parts of the system, is fully exerted, so as to make, out of so many different elements, a single whole—out of so many lives, a single life. In those situations, the nerves must act on the blood as the blood acts on the nerves.

"M. Dupuytren proved, long ago, that cutting the pneumo-gastric nerves prevents the venous from being converted into arterial blood in the lungs. Dr. Mayer, from an experiment of his own, maintains that the nervous system has an influence over the blood, not only in the capillaries, but even in the large vessels. He observed, that, whenever he tied both pneumo-gastric nerves in animals, the blood in the whole of the pulmonary system coagulated, and the colouring matter separated from the fibrine; and he took care to ascertain that these were not the consequences of death, by opening the animals the very moment they expired.

The learned and indefatigable Professor at Alfort, M. Dupuy, having lately tried upon horses the experiment of cutting the pneumo-gastric nerves in the cervical region, has ascertained that, under such circumstances, the quantity of fibrine in the arterial blood drawn from the carotid is notably diminished." 662.

M. Dupuy asserts that he found the blood entirely dissolved, in the animal whose pneumo-gastric nerves he had cut. By injecting this dissolved blood into the jugular vein of another horse he produced in the latter a gangrenous affection. These experiments, however, must be repeated before we can place dependence on them. M. Andral is firmly persuaded in his own mind, that these alterations in the blood are often primary, and precede those of the solids—consequently, that the origin of many diseases is in the blood.

"If it is true that the mass of the blood may, in certain cases, be primarily altered, it follows that the existence of general disease is not merely imaginary. In fact, when all the tissues thus receive a vitiated blood, is it not consistent with sound physiology to admit that their regular modes of vitality, nutrition, and secretion, must be more or less deeply modified? We must either admit this conclusion, or deny the influence which, according to every physiologist, the blood exerts over each solid. It may then happen that one or more organs are affected in a more decided manner than the rest, and there may thus be produced in them various lesions that are only accidental and secondary; but it is not in these lesions the origin of the affection lay; it is not on them all the symptoms depend; nor, lastly, is it to them alone we are to have recourse to throw a light upon the true nature of the disease, as well as upon the treatment proper to be pursued. Experience also teaches us, that these lesions may be either severe or slight, present or absent, similar or dissimilar, and that, notwithstanding, the disease, though presenting so many different shades in its variable symptoms depending on these lesions, does not the less exist, as it really consists in the constant symptoms depending on the state of the blood." 664.

It may be objected, that persons are seen whose blood presents all the above lesions, and who are, nevertheless, in good apparent health. M. Andral answers that such individuals are on the brink of disease--and, if any cause chances to derange the equilibrium of the system, some of the morbid phenomena resulting from impure blood are pretty sure to occur.

The diseases that seem to be connected with a morbid state of the blood, are either acute or chronic. Our author next proceeds to make some observations on each of these classes.

He has already established this fact, that, under the influence of a state of *hyperæmia*, every organ becomes excited—that death may result from this excitation—and that, in such cases, a superabundance of blood is found in all parts of the body—without any appreciable lesion of texture.

"In such cases there exists that state of pyrexia termed by nosologists inflammatory fever. But if, instead of being simply in excess, the blood contains more fibrine than ordinary, its exciting power will be still greater, and what it did in the former case merely by its increased quantity, it will do now by its alteration in quality.* Under such circumstances, is it not evident that one of the indications to be fulfilled must be to dilute the fibrine with more water? Hence the utility of administering plentifully aqueous drinks. M. Piorry has lately announced, that one of the means of preventing the formation of false membranes in croup is to gorge the patient with

* "An increase in the quantity of fibrine in the blood produces some remarkable effects on several vital actions. Thus MM. Prevost and Dumas have ascertained, that the faculty of producing heat increases in animals in proportion to the number of the globules of the blood."

water; and we know that M. Magendie has seen the symptoms of meningitis diminished by injecting water into the veins of the patient." 665.

The buff of blood, our author observes, is not the necessary result of local irritation, inasmuch as it is often found before any such process commences—simply, from the presence of a plethoric habit, or of a disposition to sanguineous congestions. It is found in pregnant women, who are so disposed to hyperæmia subsequently to parturition.

"The ancients, then, were, perhaps, in the right in admitting an *inflammatory state* of the blood, of which the phlegmasiæ of the solids were often merely the effect and external manifestation, as it were. On this inflammatory state of the blood would appear especially to depend, in certain cases, the phlegmasiæ of the membranes of the serous and articulating cavities. In the first place, we are to observe that the morbid secretion that takes place in the surface of several serous membranes when inflamed, is exactly similar, in its physical and chemical properties, to the albumino-fibrinous substance that forms the buff; and that it may be organized even within the vessels (provided there be a stagnation of the particles that form it) just as it is observed to become organized on the serous membranes on which it has been deposited. Now, if we mark the symptoms and progress of acute rheumatism, we find that very often a well-marked febrile action, with a strong reaction, but without any symptom whatever of local affection, precedes the pains. In a word, there is first an inflammatory fever, and then rheumatism. Next observe the extreme mobility of these rheumatic pains; they run along in a manner wherever the blood is distributed; the application of leeches often removes the pain from one part, but it soon shifts to another; and not unfrequently it quits the articulating tissues, and fixes upon different internal organs, producing by the derangement of their functions symptoms more or less severe. It often happens that a bleeding from a large orifice puts an end to the disease; as if, by diminishing the mass of blood, it proportionably diminished the stimulus that produced all these shifting irritations. When that occurs, in the subsequent bleedings the buffy coat becomes less apparent, and at last disappears. But if, on the contrary, the rheumatism does not yield to venesection, the buffy coat persists, and even becomes more apparent as venesection is more frequently resorted to: the serum increases while the coagulum diminishes, and yet, be the coagulum ever so small, it is nevertheless covered with a buff as long as the rheumatism continues." 667.

The above, he admits, is only matter of conjecture—but there is probably more foundation for it than for some sublime theories that have travelled far and wide in their day.

When deleterious substances, as pus, putrid matters, and poisons from the three kingdoms of Nature, have been introduced into the blood, the following phenomena are often observed, viz:—1st. The nervous centres become much affected, producing, according to the degree or nature of the affection, instantaneous death, prostration of strength, convulsions, delirium, &c. or dyspnœa, palpitation, or vomitings. 2dly. Gangrene of one or more parts. 3dly. Various sanguineous or serous exhalations. 4thly. Unusual gaseous exhalations. 5thly. Derangement of function in various organs.

"On opening the bodies of the animals that presented these different morbid phenomena, we are sometimes unable to discover any appreciable lesion: sometimes we meet with congestions or effusions of blood resembling those that had been observed during life; and, lastly, we sometimes discover greater or less alterations in the texture of the different solids. Along with these variable phenomena we always observe the two following constant ones; 1. a remarkable fluidity of the blood; and, 2. a more rapid decomposition than ordinary of the blood itself, or of the different solids it penetrates.

Where is the source of these various phenomena? Is it not evidently in the blood, into which the deleterious substances have been introduced? Now, those derangements of functions and organs produced by the experimenter, when he introduces different deleterious substances directly into the blood, are likewise those that are produced by the sting or the bite of certain animals; they are also those that take place from touching the flesh of animals that die of the plague, as well as those observed in small-pox, measles, and scarlatina, of a malignant nature, as it is called. They are the same derangements that appear in persons exposed to putrid emanations, vegetable or animal, and to miasmata from the bodies of other persons that are themselves diseased and crowded in confined places where the air is constantly receiving the infection, without being changed by ventilation. Lastly, they shew themselves also in individuals whose blood is only imperfectly or badly repaired by insufficient or unwholesome diet." 669.

The author asks what it is that we discover as the element of the disease in all these cases? Not, says he, any determinate lesion of one or more organs—for, often, no such lesion is found. It is therefore he thinks, "a vitiation of the blood by the commixture of deleterious substances—next, and consequence of such vitiation, disordered function of the nervous system—and lastly, a constant, though not always appreciable modification of the different organs by the vitiated blood."

It is to be remarked that diseases resembling the above, both in symptoms and POST-MORTEM appearances, not unfrequently occur in cases where no deleterious substance has been introduced into the blood, and where there is no direct proof that any alteration of that fluid has been the primary cause of the morbid phenomena. If, however, says M. Andral, these phenomena are perfectly identical with those which are evidently produced by vitiated blood—if, on examining the body we cannot detect here, any more than in the preceding cases, any constant lesion in the solids—and, if we always observe a certain number of fundamental symptoms, whether these lesions exist or not—what is the conclusion which we ought to draw, consistent with true logic and sound physiology?

"Certainly this, that here, as in the preceding cases, it appears that the primary cause of the disease should be referred to the blood, which, in this case has altered its nature under the influence of unknown causes, as it has in the others in consequence of the commixture of various foreign substances." 671.

Perhaps (he observes) there are cases of this kind, where the modification of the blood is only *secondary* to a modification of the nervous system. If, for instance, under the influence of a strong mental emotion, the nervous system be suddenly deranged in its functions, and ceases to exert its proper influence over the different organs in which the blood is elaborated, deposited, and receives new materials; must not that fluid itself become altered in its turn? If so, there must thence arise a number of organic and functional derangements, varying greatly according to the mode and intensity of the primitive alteration of the ennervation. In such cases, we may observe to occur sporadically, those same diseases, typhoid or other, that we have just now seen prevailing epidemically under the influence of manifest causes of infection of the blood.

"All this is, undoubtedly, I again repeat, probable, but not certain; but is there any greater degree of certainty in the opinion of those who regard all these derangements as the constant and necessary result of an acute inflammation of the stomach? I do not mean to say that this is never the case, and I am sure that no one would suppose me to entertain such an opinion. But, what I assert is, that often enough there is no proof whatever of the existence of this gastritis, that it can be admitted only by analogy, and that there is as much physiology in one hypothesis as in the other. If then, there are on all sides only more or less probable conjectures, it must be for the interests of the science that they should be all brought forward, provided that they are only given as conjectures, sufficiently founded, however, on facts and on physiological considerations, to entitle them to some share of attention. It appears to me quite certain that the theories of solidism in general, and that of irritation in particular, are insufficient to account for all the facts that have been observed. Under such circumstances what should we do? Take another position, and try what we shall gain by it." 672.

So far for acute diseases, the primary causes of which may, with some probability, be ascribed to the condition of the blood. Let us now see what the author has collected respecting chronic affections.

"When a person is in the habit of taking too much food, and that containing a great deal of azote, while at the same time his body loses little by exercise or otherwise, his blood becomes very rich in fibrine, and he acquires a disposition to those inflammatory diseases already mentioned. This is often all that is observed. In other cases, however, under similar circumstances, a superabundant secretion of uric acid takes place in the kidneys, and gives rise to the complaint called the gravel. It often happens, too, that at the same time that this acid exists in the urine in much greater quantities than usual, it occurs as a morbid secretion in several other parts of the system. It fills the joints, is deposited between the surrounding fibrous tissues, and is found in masses between the fasciculi of several muscles, in the subcutaneous cellular tissue, and even in the spongy extremities of the bones. I have found deposits of uric acid in all these parts simultaneously, in the body of a patient that died at *La Charité*, whose case is to be found described in the inaugural dissertation of Dr. Fauconneau Dufresne. In such cases, it appears that this uric acid, which thus appears in all parts of the body, and which we know from chemistry to be one of the most highly azotized proximate animal principles, is formed in excess in the blood under the influence of a strongly azotized diet; and that it is separated from it by its natural emunctory, as well as in the texture or on the surface of other organs. Accordingly, as Magendie remarks, the best way to put a stop to this superabundant secretion of uric acid is to change the diet of the person affected, and give him food containing as little azote as possible. Now, what is the prominent feature in all this? The modification in the composition of the blood by the food, and the production of disease in consequence. According then, to this view of the subject, there is something more in affections termed gouty, than a merely local irritation of an organ; the latter is only a secondary phenomenon, and we have other indications to fulfil than that of combating the pains in the joints by blood-letting. This theory is not incompatible with the fact that gravel and the different other deposits of uric acid are sometimes observed in persons who are far from living on such diet as I have just described; for we may conceive that independently of any influence of diet, the azote of the blood may become spontaneously predominant, and consequently a greater quantity of uric acid be formed: it is however well known that this is not the most common case." * 674.

In the valuable work of Professor Dupuy on tuberculous affections, we find a fact that bears upon those just mentioned. He states that, in several cows, whose lungs presented abundant deposits of carbonate of lime, it was ascertained that the milk also had a much greater quantity than usual, of the same salt. Hence M. Andral thinks it probable that, at that age when phosphate of lime is frequently deposited in different organs, the cause may be found in superabundance of that salt in the blood. Under the influence of various agencies, as those of food and air, we find the blood altered, and therefore most probably the source of those diseases which arise under such circumstances. In times of scarcity, when a large portion of the population is forced to live on scanty or unwholesome provisions, dropsical dispositions have been al-

* "It has been lately proved by the experiments of M. Edwards, that animals expire less azote during the cold season. Is not this one of the causes that may contribute to the superabundant formation of uric acid in cold, damp countries? This would be one secretion supplying the place of another."

ways observed to prevail. Dropsy, however, often shews itself spontaneously. In the Clinique Medicale, the author has recorded the history of some dropsical patients, on the examination of whose bodies no appreciable alteration of solids could be detected; but, in whom the blood was nothing but serum—at least it was devoid of colouring matter throughout; and if it contained any fibrine it had lost the power of coagulating.

"The dropsy accompanying such a state of the blood may appear either in persons who were previously in good health, or in those exhausted by long sickness. The first sort seem to have been in a manner disposed to it, from their pale, dead colour, their soft flesh, and the habitual state of semi-infiltration of their subcutaneous cellular tissue. If we apply leeches to their skin, in place of true blood, we often observe only a reddish serum issuing from the bites; and as, in such cases, it is not possible for coagulation to take place, it is sometimes not without the greatest difficulty we can succeed in stopping it. Again, if we produce any irritation, there is but little appearance of redness; but a rapid accumulation of serum in the cellular tissue subjacent to the irritated part takes place. Thus, in this case, the result of the irritation is determined by the state of the blood; a very evident fact, and of some importance with respect to what may be drawn from it. Compare the bloodless skin of such individuals with the brown firm skin of stout healthy persons, with the bright rosy tint that marks the state of plethora; and the three, with the habitually yellow tinge in persons of a bilious temperament, as it is called, who are yet in good health; compare them, I say, and it will be evident that we cannot consider the fluid traversing the cellular tissue of the skin in these different individuals, as possessing the same properties, as containing the same principles, and as capable of acting similarly on the tissues; now, this fluid is, after all, but a part of the whole mass of the blood, and from the state of that part we can judge of the state of the whole." 678.

In the disease termed scurvy, there is such an evident deterioration of the blood, as to prove a stumbling-block to the exclusive solidists in all times. In most instances the food, or air, or both, are bad before the phenomena of scurvy appear. In other cases the scurvy rises without antecedent bad food; but even in such instances, will it be denied that the blood is vitiated, though we cannot discover the primary cause? In a majority of cases we are ignorant of the primary causes that induce lesions on the solids themselves. If, too, we find that in scurvy there are numerous local affections, such as effusions of blood, tumefaction of the gums, ulcers, splenic congestions, dyspnœa and palpitations, &c. which local affections, though greatly differing from each other, are evidently dependent upon one general affection, is it not likely that scurvy is not the only disease thus circumstanced? Is it not, says he, natural to suspect that, in every case where we see a great many organs simultaneously affected in their nutrition—

where they are also simultaneously the seat of morbid secretions, more or less similar in their nature—and where these alterations appear in the same order and manner in numbers of persons—they are not merely a chance assemblage. It is more natural, he observes, to suspect that there is in the system a pre-existing morbid condition that reveals its existence by these various local affections—so that to disperse the latter, we must attack the former.

"Now, it is evident that this general morbid condition, of which every organ feels the influence, can hardly consist in any thing else than a modification of one or other of the two equally general systems, the sanguineous and nervous, which give life and support to each organ. Every one is acquainted, for instance, with the very striking features that characterize the scrofulous constitution, and every one must allow, on ever so little reflection, how impossible it is to confine such a state to any one particular part, whether the health still exist, or have been deranged by the alterations of nutrition taking place on all sides. In such cases, as there is no escaping the influence of the morbid condition which prevails over the whole system, and is present every where in the blood, every process of nutrition will be altered, and every secretion modified; every hyperæmia accidentally produced will present a peculiar character in its symptoms, progress, duration, termination, and in the effects of therapeutic agents on it; and every process of suppuration will furnish a fluid of equally peculiar characters. At the same time, there is not a single one of these alterations that may not be, in other cases, purely and simply a local affection; such is the case, for instance, with pulmonary tubercles. That, however, is precisely what it is important to distinguish; and I have already had occasion to establish this distinction when treating of tubercles.

I have attempted to demonstrate, above, that there must be an alteration in the qualities of the blood, when there is an alteration in the secretions. In consequence of the vitiation of these latter, there appear different morbid states in the production of which the qualities of the blood bear an important part. This is what occurs when the liver, for instance, no longer abstracts from the blood in proper quantities the materials of the bile, they being either formed more abundantly than usual in the blood, or the liver, whether appreciably altered or not in its texture, having lost the power of secreting them. They then remain in the blood, and thence results a yellow tinge, of greater or less intensity, in the skin and several other tissues. They may also escape from the blood with the elements of other secretions, and are to be found in the sweat, urine, lymph of the thoracic duct, fluid furnished by the mucous membranes, and that exhaled on the surface of the serous membranes. It even occurs, sometimes, that the bile forms deposits in the parenchyma of different organs; where it is found accumulated, in the same manner as purulent collections are found in others. The resinous matter of the bile has sometimes been found in these various fluids and solids, but its yellow colouring matter is of much more frequent occurrence. When the bile has once *passed into the blood*, to use the common expression, (which here again, happens to be consonant with the science, is it not reasonable to admit that that fluid, being altered by its unusual mixture with the elements of the bile, can no longer exercise its regular influence over the different organs to which it is distributed? Hence must arise different series of symptoms, according, 1. to the state in which these organs are; and, 2. according as the mixture of the bile, or, at least, of its elements, with the blood, is more or less intimate, more or less prolonged, and more or less abundant. I may, perhaps, be mistaken; but it seems to me that such a cause is very capable of producing some of those febrile states that have been denominated bilious fevers, a generic expression answering to more than one kind of morbid state. In fact, let us consider, in a number of individuals, how the symptoms of this disease are grouped together, and how they succeed each other; let us observe the very remarkable yellow tinge of the face and conjunctiva, the slight icteric suffusion which sometimes affects more or less the rest of the cutaneous surface, the yellow tinge of the various excreted fluids, such as the urine, the mucus of the nasal fossæ, and the expectoration, the yellow coat of the tongue, and the very abundant bilious evacuations that often take place both above and below at the same time; sooner or later after the appearance of this kind of bilious plethora, different functions become disordered, and at last the fever is kindled. Now, where are we to suppose the cause of all this to reside? Is it in the irritation of an organ, of the digestive canal, for instance? The fact of the matter is, that the existence of such irritation can often be admitted only by hypothesis. Before the fever begins, and while there is yet only a bilious state, to use the phrase of some writers, should we attempt to remove it by blood-letting? Experience has proved its inefficacy in such cases. If it was proved, on the contrary, that such medicines as evacuate the intestinal canal, when properly employed, restore the patient to health, we might explain their success by the greater activity they give the secretion of the liver, thereby producing a more complete depuration of the blood, and a cessation of the bilious symptoms, as they are called. I have had opportunities of examining the bodies of different persons that had died of jaundice after suffering under the disease for several months; they had fallen by degrees into a state of marasmus, and at last went off insensibly, without having ever presented symptoms of irritation in any one organ. In many of these cases, I could not discover any appreciable lesion in the liver, or other organs. What, then, was the cause of the disordered functions, emaciation, and death? Was it the prolonged infection of the blood by the bile?" 684.

Every one knows what serious symptoms appear in animals when their ureters are tied—and in man when any cause suspends the excretion of urine. In either case the blood becomes changed, and we observe that assemblage of symptoms comprised under the generic term of adynamic, putrid, or ataxic fever. M. Andral appears inclined to support the ancient doctrine of milk fever and milk depôts, observed in females where the mammary secretion is interrupted or suppressed. The following case is adduced from M. Graefe's journal.

"A miller's wife was delivered of a child, which she suckled herself. On the eighth day after her confinement, while in perfect health, the crash occasioned by the fall of a mill wheel frightened her so much, that her milk was totally suppressed. A state of constant febrile excitement then ensued, which degenerated into a tertian ague, during the course of which her legs became œdematous, and at the end of three weeks she became affected with anasarca and ascites. Three weeks after, as the dropsy did not diminish, recourse was had to paracentesis, and a bucket of fluid drawn off, which resembled whey, exhaled an acidulous odour, and, upon being boiled with dilute sulphuric acid, coagulated, and afforded a substance exactly resembling caseum. Six weeks afterwards, the peritoneum being again filled, a second puncture was made, which gave issue to a fluid of a greenish yellow, without the least trace of caseum. The patient recovered." 686.

The above and other facts, he thinks, appear to demonstrate the possibility of the formation of one of the most important principles of the milk in other parts of the system, besides the mammæ. In the case just quoted, the succession of the symptoms was very remarkable. A mental impression suspends the lacteous secretion. This suspension is succeeded by ague—and this again by dropsy, the fluid of which contained a matter resembling caseum.

The author then adverts to the influence of the composition of the fluids over that of the secretions, and reminds the reader that Magendie, by changing the food of different animals, and consequently modifying their blood, made the urine and the bile of some of the carnivorous animals resemble those of the herbivorous.

"Thus, then, when the nature and the proportion of the constituent principles of the blood have undergone some of the changes already described, the result must be more or less appreciable modifications in the qualities of the secreted fluids, which may play a more or less important part in the production of certain morbid states. Thus it is easy to conceive, that if many of the alterations in the saliva, bile, urine, serum, &c. are the direct result of a lesion of the organ in which these fluids are elaborated, there are others, independent of the state of the secreting organ, and connected with an alteration of the common fluid from which they all emanate. If that is the case, we may go still farther; and since it appears that most of the morbid productions are deposited in the cellular tissue, in place of the small quantity of serum that usually lubricates it, we may ask if they also may not be accounted for by a vitiated state of the blood; without, at the same time, meaning to assert, as I have already explained, that many of them may not likewise result from a purely local alteration in the part where they are developed. In fact, what are these accidental productions but substances of various descriptions which are deposited in the framework of each organ, that is to say, in its cellular tissue, in place of its natural secretion? Now, we can only conceive two reasons for their appearance; either the blood on its arrival at an organ is wrongly elaborated by the nutritive parenchyma of that organ, or else it is altered before its arrival there. But, there are many cases in which there is no proof of there having been any thing wrong in the structure or action of the organ before the appearance of the morbid deposit; and we may with especial reason doubt the previous existence of any fault in the part, when a great many organs simultaneously become the seat of similar accidental productions." 689.

We have now brought this important chapter of M. Andral's work to a close. By many, the humoral pathology of the author will be treated with contempt—by others it will be read with interest, and, we think, with profit. It is not improbable that a practical basis will be laid, for the distinction of fevers dependent on the state of the blood from those where the nervous system is the primary seat of the disease. In the large and important class of malarious fevers, it is impossible not to suppose that a poison is taken into the circulation, even if the first impression of that poison be evinced in the sentient system. Thus a certain dose of malaria, whether from the earth or from a crowded population, vitiates the blood and shocks the nervous system, inducing a train of exertions in the constitution, whether in periodical paroxysms or uninterrupted struggles, till the poison is exhausted, or till it destroys the living machinery of the system. But, however the cause of fever may act in the first instance—whether on the solids or on the fluids, we cannot but admit that the mass of blood generally evinces some kind of deterioration, and that this vitiation plays in the drama of the malady, a very important part.

